# Er: YAG laser-activated 17% EDTA with side-firing tip preserves root canal dentin integrity: a micro-CT study

**DOI:** 10.1007/s10103-026-04800-z

**Published:** 2026-01-26

**Authors:** Sharonit Sahar-Helft, Noy Pinto, Aharon Dakar, Coral Helft, Doron Steinberg, Yaniv Mayer

**Affiliations:** 1 Department of Endodontics, Rambam Health Care Campus, Haifa, Israel; 2 Faculty of Medicine, Technion – Israel Institute of Technology, Haifa, Israel; 3 Faculty of Dental Medicine, Hebrew University of Jerusalem, Jerusalem, Israel; 4 Sagol School of Neuroscience, Tel Aviv University, Tel Aviv, Israel; 5 Department of Periodontology, Rambam Health Care Campus, Haifa, Israel

**Keywords:** Er:YAG laser, Side-firing spiral tip, 17% EDTA, Dentin thickness, Canal volume, Microcrack formation

## Abstract

**Purpose:**

The primary objective of endodontic therapy is to eliminate pathogenic biofilms from the intricate root canal system. Despite significant advancements in instrumentation and conventional irrigation, these methods often fail to adequately disinfect anatomically complex areas, leading to treatment failure. The integration of Er: YAG laser-activated irrigation (LAI) offers a promising adjunctive method to enhance disinfection through photoacoustic and cavitation effects. However, concerns regarding potential iatrogenic damage, such as a reduction in dentin thickness or microcrack formation due to thermal stress, persist. This study aimed to evaluate the safety of LAI with an Er: YAG laser using a side-firing spiral tip in combination with 17% EDTA by assessing potential structural changes in dentin thickness, canal volume, and microcrack formation, all assessed by micro-computed tomography (micro-CT) analysis.

**Methods:**

Twenty single-rooted human teeth were accessed and prepared using the ProTaper Ultimate rotary system up to the F3 size. The specimens were scanned using micro-CT before the laser treatment to obtain baseline measurements of dentin thickness and canal volume. Teeth were then treated with Er: YAG laser LAI using a side-firing spiral tip at parameters of 1.5 W, 150 mJ, and 10 Hz for 60 s, with 17% EDTA as the irrigant. After the laser treatment, a second micro-CT scan was performed. Pre- and post-treatment micro-CT data were superimposed and analyzed for changes in dentin thickness, canal volume, and the presence of microcracks.

**Results:**

The micro-CT analysis revealed no statistically significant difference between the pre- and post-treatment measurements of dentin wall thickness or canal lumen volume at the cervical, middle, and apical thirds of the root. The average change between pre- and post-treatment measurements did not exceed 0.05 mm, except for the palatal aspect in the middle part of the root. Furthermore, a careful examination of all specimens showed no evidence of microcrack formation following the LAI protocol. These findings demonstrate the safety of this protocol in preserving tooth structure.

**Conclusion:**

Our findings demonstrate that Er: YAG LAI with a side-firing spiral tip, when applied with the specific parameters tested (150 mJ, 10 Hz, 60 s), does not cause detectable structural damage to root dentin, supporting its safety profile within the context of this ex vivo study. These results suggest this protocol may be a safe adjunct for root canal disinfection, though in vivo validation and comparison with alternative activation methods are needed.

## Introduction

Endodontic therapy is among the most complex procedures in contemporary dental practice. The primary objective is the elimination of pathogenic biofilms from the intricate root canal system, as microbial persistence is closely associated with treatment failure and reinfection [1, 2]. Although advancements in instrumentation and irrigation protocols have improved treatment outcomes, conventional methods are often limited in their ability to clean anatomical regions inaccessible to mechanical preparation, such as isthmuses, apical deltas, and lateral canals [2]. This challenge has driven the development of adjunctive disinfection methods capable of enhancing irrigant penetration and efficacy.

One of the most significant innovations in this regard is the integration of laser technology into endodontic protocols. The erbium-doped yttrium aluminum garnet (Er: YAG) laser, with its wavelength of 2,940 nm, has optimal absorption in water and hydroxyapatite, making it particularly suitable for root canal applications. Its ability to disrupt biofilms and smear layers while minimizing thermal damage to dentin has been well documented [1]. Recent reviews, systematic reviews, and consensus guidelines have confirmed that laser-activated irrigation (LAI) provides superior disinfection compared with conventional and ultrasonic irrigation techniques, with enhanced antimicrobial efficacy and improved debris removal from complex anatomies [3–5].

Early laser delivery systems, however, were not without limitations. Flat-ended tips emitted energy apically, generating vapor bubbles that could extend beyond the fiber end, raising concerns about irrigant extrusion and periapical tissue injury [6–8]. To address these risks, side-firing spiral tips were developed, designed to emit energy laterally and incorporate a terminal occlusion to prevent apical extrusion. These tips also optimize hydrodynamic activation by producing shock waves and turbulent flow that distribute irrigants effectively into anatomical complexities [9].

The combination of Er: YAG laser activation with chelating agents such as 17% EDTA has been shown to further enhance smear layer removal and biofilm disruption [10, 11]. Recent investigations have also characterized the mineral composition changes following Er: YAG LAI with EDTA and sodium hypochlorite, demonstrating predictable alterations in the calcium-to-phosphorus ratio without compromising structural integrity [12]. Despite these advantages, concerns remain regarding the potential adverse effects of laser energy on dentin integrity. High energy settings or prolonged exposure may result in temperature elevation, structural microcracks, and weakening of the root canal wall, potentially predisposing teeth to vertical root fracture [13–15].

Recent advances in micro-computed tomography (micro-CT) have enabled non-destructive, high-resolution assessment of dentin thickness, canal volume, and microcrack formation [16–18]. This technology provides an accurate method to evaluate the safety of innovative irrigation protocols.

Therefore, the aim of this study was to investigate the structural safety of Er: YAG laser–activated irrigation using a side-firing spiral tip in combination with 17% EDTA on dentin thickness, canal volume, and microcrack formation, as assessed by micro-CT analysis. This study specifically evaluates the potential for iatrogenic structural damage, which is a prerequisite for clinical application and complements existing antimicrobial efficacy data.

## Materials and methods

### Tooth Preparation

Twenty single-rooted human teeth were selected for this investigation following ethical approval (Helsinki approval number 040617-HMO). Clinical trial number: not applicable. Single-rooted teeth were selected to minimize anatomical variability and ensure standardized micro-CT measurements and consistent laser tip positioning throughout the canal system. Standardization of root length was achieved by sectioning specimens to 18 mm at the coronal aspect using a diamond disc mounted on a straight handpiece, thereby preserving the natural apical anatomy including the apical constriction and foramen. This approach maintained clinically realistic apical conditions for evaluating laser activation effects. Radiographic verification confirmed accurate working length determination for each specimen. The working length was established as 1 mm short of the radiographic apex, confirming preservation of the apical constriction. Initial coronal access was established using Gates Glidden burs #2 and #3 (Dentsply Sirona Inc.), followed by negotiation with a #10 K-file until apical patency was confirmed. Sequential instrumentation commenced with #15 and #20 K-files, progressing to ProTaper Ultimate rotary instruments (Dentsply Maillefer, Ballaigues, Switzerland) through F3, similar to the routine clinical procedure. Final apical preparation was completed using #30 and #40 K-files to ensure adequate chemo-mechanical debridement. Between each instrument, canals were irrigated with 10 mL double-distilled water (DDW) delivered via a 25-gauge needle (0.5 × 16 mm) positioned 2 mm short of the working length. Apical patency was maintained throughout preparation using a #10 K-file.

## Laser specifications

Er: YAG laser system (Light Instruments, Yokneam, Israel) equipped with a lateral-emission helical endodontic tip (LiteTouch™; Light Instruments, Yokneam, Israel) was utilized in this investigation. The endodontic tip features a peripheral helical aperture design that is pliable, tubular, tapered, and exhibits a circular cross-sectional geometry.

The endodontic tip was advanced through the root canal system until reaching the periapical region. These parameters (150 mJ, 10 Hz, 60 s) were selected based on prior thermal safety data showing temperature increases below 5 °C at all root surface sites—well below the 10 °C threshold for tissue damage. Temperature elevations exceeding 10 °C can cause irreversible damage to periodontal ligament cells, induce root resorption, compromise bone vitality, and potentially affect residual pulpal tissue in multi-visit treatments. By maintaining temperature increases below 5 °C, these parameters minimize thermal injury risk to periapical structures while enabling effective hydrodynamic activation. This study evaluated whether these thermally safe parameters also preserve structural integrity. The operational protocol consisted of reciprocating tip movement in 1–2 mm increments along the coronal-apical axis, combined with the irrigation protocols described below.

Root canal assessment was conducted both prior to Er: YAG laser activation following 17% EDTA treatment and subsequent to laser therapy. Evaluation encompassed three distinct anatomical regions (cervical, middle, and apical thirds) across four canal wall surfaces (buccal, lingual, mesial, and distal orientations).

## Irrigation protocol during laser activation

Prior to laser activation, root canals were filled with 2 mL of 17% EDTA solution (pH 7.4, MD-Cleanser, Meta Biomed, Korea) delivered via syringe and 30-gauge needle to the full working length. During the 60-second laser activation period, the canal remained filled with EDTA solution, with no additional irrigant flow (static fill protocol). The laser tip was activated while performing continuous reciprocating movements as described above, ensuring hydrodynamic activation throughout the canal system. After laser activation, canals were flushed with 5 mL of double-distilled water to remove debris and residual EDTA.

The 60-second activation time was selected based on multiple considerations: This duration is consistent with clinical protocols reported in recent LAI literature [3, 4]; and it represents a practical duration for clinical application while ensuring adequate hydrodynamic activation of the irrigant throughout the canal system, including anatomically complex regions such as isthmuses and lateral canals.

## Micro-Computed tomography (µCT) imaging

Teeth were measured after the enlargement, with endodontic files, before the laser activity, and after laser irradiation. Teeth underwent scanning via µCT40^®^, Scanco Medical, Brüttisellen, Switzerland at 10 μm isotropic nominal resolution, 70 kV energy, 114 µA intensity, and 1,000 projections at 200 millisecond integration time. Each tooth was scanned at three levels: cervical, middle, and apical thirds. All scans were analyzed in axial (cross-sectional) view. At each level, dentin thickness was measured at four points around the canal wall: buccal, palatal, mesial, and distal. Canal lumen diameter was measured along two perpendicular axes: buccal-palatal (B-P) and mesial-distal (M-D).

## Micro-CT image registration and measurement protocol

Pre- and post-treatment micro-CT datasets were superimposed using a voxel-based rigid registration algorithm within the manufacturer’s analysis software (Scanco Medical Evaluation Program, Scanco Medical, Brüttisellen, Switzerland). The registration process was performed as follows: First, the external root surface geometry, which remains unchanged during irrigation procedures, was used as the primary reference structure. The software automatically identified common anatomical landmarks between pre- and post-treatment scans, including the apex, cementoenamel junction, and external root contours. The registration algorithm then iteratively optimized the alignment by minimizing the sum of squared differences between corresponding voxels of the two datasets.

Registration accuracy was verified through three quality control measures: (1) visual inspection of overlay quality by examining the alignment of the external root surface in multiple planes (axial, sagittal, and coronal), (2) calculation of root mean square (RMS) error between registered surfaces, with acceptable values defined as < 10 μm, and (3) independent verification by a second observer for 10 randomly selected specimens. All specimens met the quality criteria, confirming reliable superimposition.

For standardized measurements, the long axis of each root was first established by identifying the apical foramen and the coronal pulp chamber floor. The root was then divided into three equal segments (cervical, middle, and apical thirds) along this axis. At each level, cross-sectional images perpendicular to the long axis were extracted at the midpoint of each third. The canal wall boundary and external root surface were automatically segmented using density threshold values (enamel/dentin: 1200–3000 mg HA/cm³; canal space: 0–400 mg HA/cm³), with manual correction applied when necessary.

Within each cross-sectional image, four measurement points were established using an angular coordinate system centered on the canal centroid: buccal (0°), mesial (90°), palatal (180°), and distal (270°). At each point, dentin thickness was calculated as the shortest Euclidean distance from the canal wall to the external root surface. Canal lumen diameter was measured along two perpendicular axes: buccal-palatal (B-P) and mesial-distal (M-D), defined as the maximum distance across the canal lumen at each orientation.

All measurements were performed by a single calibrated operator. To assess intra-operator reliability, 20% of specimens (*n* = 4) were randomly selected and re-measured after a 2-week washout period. Intraclass correlation coefficients (ICC) for dentin thickness and canal diameter measurements were 0.97 and 0.96, respectively, indicating excellent reliability. The mean difference between repeated measurements was 0.008 mm (95% CI: −0.012 to 0.028 mm), well below the threshold for clinical significance.

Pre- and post-treatment micro-CT scans were systematically evaluated for microcracks using multiplanar reconstruction at 0.1 mm intervals. Microcracks were defined as new linear defects extending from the canal wall. Suspected cracks were differentiated from anatomical structures by tracing through consecutive slices. 20% of specimens were independently reviewed.

### Quantification and statistical analysis

All measurements of root canal diameter were recorded as raw values in the ‘Before’ and ‘After’ laser groups. For each region (Cervical, Middle, Root), the ‘Before’ values serve as baseline control. The mean of the ‘Before’ values was calculated and used to normalize the corresponding ‘After’ values, which were expressed as percentages relative to baseline (normalized value x100).

All data were analyzed with GraphPad Prism v.8 (GraphPad Software, San Diego, CA, USA). Data are presented as mean ± SEM.

Within-specimen comparisons between pre- and post-treatment measurements were conducted using the paired Student’s t-test. Statistical significance was set at α = 0.05. Results are presented as mean ± standard error of the mean (SEM) with 95% confidence intervals (CI). Effect sizes were calculated using Cohen’s d to assess the magnitude of any observed differences.

### Sample size calculation

An a priori power analysis was conducted using G*Power software (version 3.1.9.7, Heinrich Heine University, Düsseldorf, Germany) to determine the minimum sample size required to detect clinically meaningful changes in dentin thickness. The estimation of effect size and standard deviation was based on previously published micro-CT studies of endodontic procedures.

Based on micro-CT studies by Ceyhanli et al. [14] and Stern et al. [16] reporting dentin thickness changes of 0.04–0.08 mm and measurement precision of ± 0.03 mm, we defined a minimal clinically significant difference of 0.05 mm. Standard deviation was conservatively estimated at 0.03 mm based on published data from Stern et al. [16], which reported variability ranging from 0.025 to 0.035 mm for micro-CT dentin measurements.

Using a two-tailed paired t-test with α = 0.05, power = 0.80, effect size = 0.05 mm, and SD = 0.03 mm, the minimum required sample size was calculated as *n* = 18 teeth. To account for potential specimen loss or technical failures, we enrolled 20 teeth, providing adequate statistical power with a safety margin.

## Results

Micro-CT imaging was used to quantify morphological changes in dentin thickness and canal lumen diameter following Er: YAG laser-activated irrigation with a side-firing Endo tip in combination with 17% EDTA. Measurements were performed on axial sections at three root levels: cervical, middle, and apical. At each level, dentin thickness was measured at the buccal, palatal, mesial, and distal walls, while canal lumen diameter was assessed along the buccal-palatal (B–P) and mesial-distal (M–D) axes.

## Dentin thickness

**Fig. 1:**
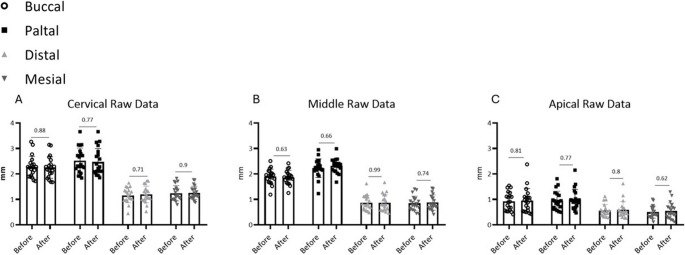
Dentin width measurements before vs. after laser scanning (raw data (mm)); four aspects of measurements (Buccal, Palatal, Distal, Mesial) (**A**) Cervical part of root (**B**) Middle part of root (**C**) Apical part of root

**Fig. 2:**
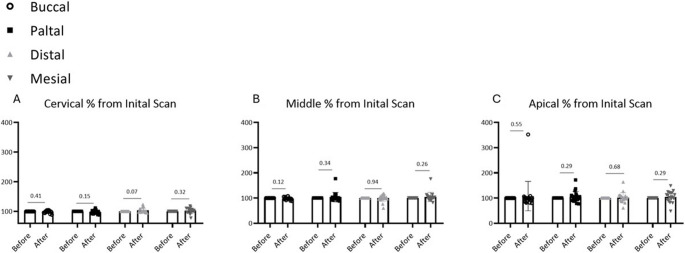
Dentin width measurements before vs after laser scanning (“after” normalized to “after” (%)); four aspects of measurements (Buccal, Palatal, Distal, Mesial) (**A**) Cervical part of root (**B**) Middle part of root (**C**) Apical part of root

Quantitative comparison before and after irradiation revealed no statistically significant reduction in dentin wall thickness at any of the evaluated root levels (Fig. 1). No pattern of directional loss (e.g., preferential thinning in a specific aspect) was observed (Fig. 2). The average change (‘delta’) between pre- and post-laser measurements did not exceed 0.05 mm at any location, except for the palatal wall in the middle third of the root, which showed a mean change of 0.052 mm. (Table 1). In addition, standard deviation values reflect there was no big difference between teeth inside the group, which means “delta” values were relatively redundant, and results are reliable. Mean differences (post-treatment minus pre-treatment) across all measurement sites ranged from − 0.012 mm to + 0.018 mm (95% CI: −0.028 to + 0.034 mm), with effect sizes (Cohen’s d) ranging from 0.08 to 0.15, indicating negligible practical significance. All p-values exceeded 0.05 (range: 0.32 to 0.89), confirming no statistically significant changes in dentin thickness.

**Table 1:** Values represent the mean difference (delta) between post-treatment and pre-treatment measurements (post minus pre) ± standard deviation (SD) in millimeters. Measurements were taken at four aspects of the Canal wall (buccal, palatal, distal, mesial) and along two perpendicular axes for Canal lumen diameter (bucco-palatal and mesio-distal radii) at three root levels (cervical, middle, apical)

	Buccal	Palatal	Distal	Mesial	Bucco-Palatal Radius	Mesio-Distal Radius
Cervical average (mm ± SD)	0.019 ± 0.0979	0.0475 ± 0.1527	−0.0352 ± 0.0903	−0.0103 ± 0.1153	−0.0269 ± 0.0730	−0.01185 ± 0.0590
Middle average (mm ± SD)	0.049 ± 0.1087	−0.05525 ± 0.2550	−0.0023 ± 0.0729	−0.0168 ± 0.8489	0.0046 ± 0.0717	−0.02175 ± 0.0495
Apical average (mm ± SD)	0.0477 ± 0.0689	−0.00525 ± 0.0688	0.00785 ± 0.0283	−0.02435 ± 0.0688	−0.0001 ± 0.0220	−0.01185 ± 0.018

### Canal lumen diameter

**Fig. 3:**
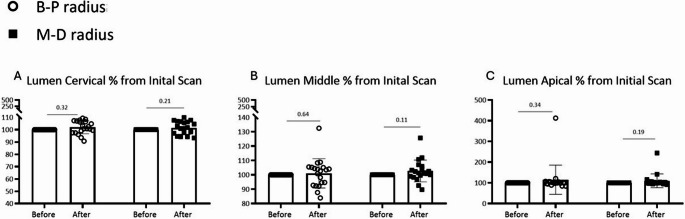
Lumen diameter (radius) measurements before vs. after laser scanning (raw data (mm)); two aspects of measurements (Buccal-Palatal (B-P) Distal-Mesial(M-D)) (**A**) Lumen measurement at the cervical part of root (**B**) Lumen measurement at the middle part of root (**C**) Lumen measurement at the apical part of root

**Fig. 4:**
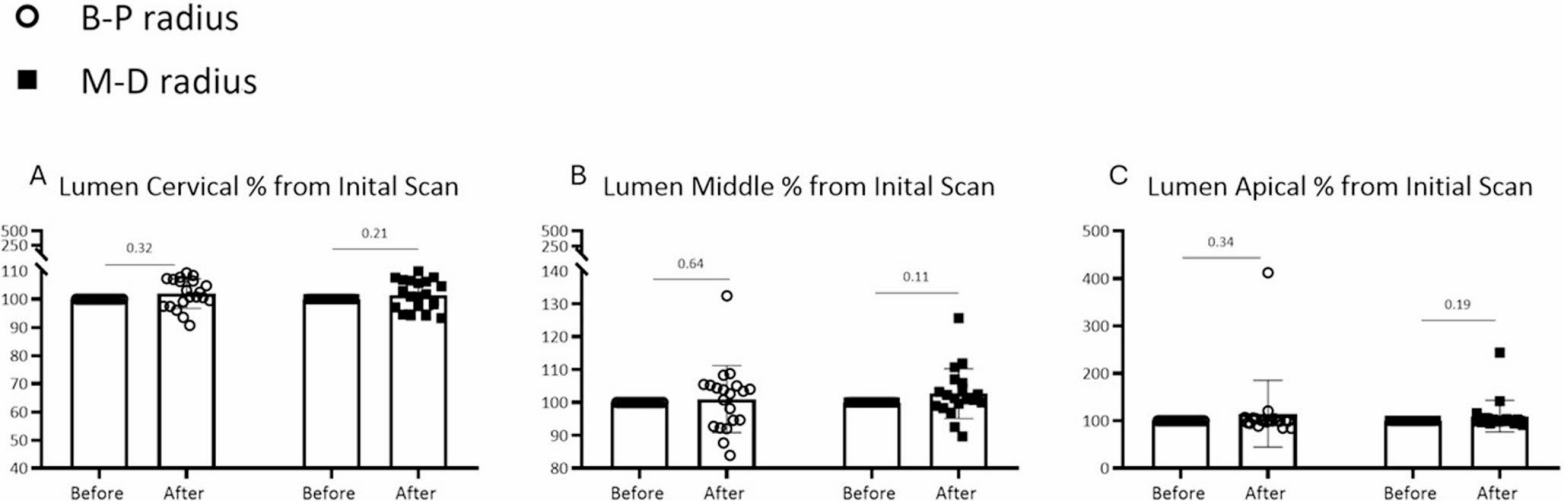
Lumen diameter (radius) measurements before vs after laser scanning (“after” normalized to “after” (%)); two aspects of measurements (Buccal-Palatal (B-P) Distal-Mesial(M-D)) (**A**) Lumen measurement at the cervical part of root (**B**) Lumen measurement at the middle part of root (**C**) Lumen measurement at the apical part of root

Similarly, canal lumen dimensions showed no significant differences between pre- and post-treatment scans (Fig. 3). The percentage change in lumen diameter was negligible across all root levels and orientations (Fig. 4).

Mean differences in canal lumen diameter ranged from − 0.008 mm to + 0.011 mm (95% CI: −0.019 to + 0.025 mm), with effect sizes (Cohen’s d) < 0.10 for all measurements, indicating trivial differences. No comparisons reached statistical significance (all *p* > 0.05).

### Microcrack formation

Systematic examination of all pre- and post-treatment micro-CT scans revealed no evidence of microcrack formation in any of the 20 specimens following Er: YAG laser-activated irrigation. Multiplanar reconstruction and slice-by-slice analysis at 0.1 mm intervals confirmed the absence of new linear defects extending from the canal wall toward the external root surface. All anatomical structures present in pre-treatment scans (including accessory canals and natural anatomical variations) remained unchanged in post-treatment imaging, with no additional crack-like features observed at any root level (cervical, middle, or apical thirds).

## Discussion

This study evaluated the structural safety of Er: YAG laser-activated irrigation using a side-firing spiral tip in combination with 17% EDTA by assessing dentin thickness, canal volume, and microcrack formation through micro-CT analysis. The results demonstrated that this protocol did not cause significant changes in dentin wall thickness or canal lumen dimensions across cervical, middle, and apical thirds, with no evidence of microcrack formation observed in any specimen.

Our micro-CT analysis revealed no statistically significant reduction in dentin wall thickness at any root level, with mean changes remaining below 0.05 mm at all measurement sites except the palatal wall in the middle third (0.052 mm). These findings align with morphological safety data from previous Er: YAG laser studies while demonstrating superior preservation compared to alternative activation methods. Arumugam et al. [19], Fontana et al. [20], and Matsumoto et al. [21] reported morphological alterations following Er: YAG irradiation, including dentinal tubule opening, but similarly documented no evidence of charring or cracking under controlled parameters (< 200 mJ). Marraccini et al. [22] evaluated dentin morphology after Er: YAG laser irradiation and confirmed absence of thermal damage when appropriate parameters were used. Our results extend these findings by demonstrating that even with continuous 60-second activation in the presence of EDTA, structural integrity is maintained when using a side-firing tip configuration. In contrast, other studies have indicated that excessive fluence or prolonged exposure may result in thermal damage and microcrack formation [13, 15].

The preservation of dentin thickness and absence of microcrack formation observed in this study have important clinical implications. Vertical root fracture represents one of the most serious complications in endodontics, with microcracks serving as potential initiation sites for catastrophic failure [11]. Previous studies have documented microcrack formation following mechanical instrumentation and ultrasonic irrigation, particularly in the apical third where dentin is thinnest [23]. The absence of iatrogenic damage in our study suggests that Er: YAG LAI with appropriate parameters may offer a safer alternative for irrigation activation compared to ultrasonic methods, which have been associated with uncontrolled dentin removal. Our findings demonstrate that the tested LAI protocol preserves structural integrity, supporting its potential for safe clinical integration. However, long-term studies evaluating dentin fatigue properties and fracture resistance following LAI treatment are needed to confirm these benefits.

When compared to alternative irrigation activation techniques, our results suggest distinct advantages of laser activation with side-firing tips. Conventional syringe irrigation suffers from the vapor lock effect [24], where trapped air prevents irrigant penetration beyond 1–2 mm past the needle tip [5], severely limiting apical disinfection. Both ultrasonic and laser activation overcome this limitation through fluid agitation, but their mechanisms and safety profiles differ substantially. Ultrasonic irrigation requires careful handling to prevent complications such as instrument deformation, instrument separation, or extrusion of irrigants into periapical tissues [5]. In contrast, laser-activated irrigation uses short pulses of energy that create bubble formation in specific areas without building up excessive pressure at the apical region [23].

Earlier laser activation protocols that employed flat-ended tips raised concerns due to apically directed energy and bubble extension beyond the tip end, which increased the risk of irrigant extrusion [6–8]. The introduction of side-firing spiral tips was designed to overcome these limitations, providing lateral energy emission and terminal occlusion, thereby reducing risks of apical extrusion while improving hydrodynamic activation [9]. The absence of morphological alterations in the present study supports the safety of this newer delivery system.

The synergistic use of Er: YAG activation with 17% EDTA is supported by evidence showing enhanced smear layer removal and biofilm disruption [10, 11]. Although the present study did not directly evaluate antimicrobial effects, the absence of dentin damage under these conditions supports the safe integration of this protocol into clinical endodontic practice, contrary to the findings [25] UI is highly dependent on the power intensity of the device, which shows uncontrolled dentin removal with UI, resulting from file-to-wall contact in apical third. Preserving dentin structure is of particular importance, as microcracks may predispose to vertical root fractures, a complication with serious clinical consequences [13]. Several Er: YAG studies report improved smear layer and debris removal, deeper irrigant penetration, and enhanced biofilm disruption in root canals, especially with photoacoustic activation modes used alongside NaOCl and EDTA [26–28]. However, most existing Er: YAG studies are in vitro or ex vivo, use heterogeneous irradiation parameters, and rarely assess in vivo temperature changes, microcrack formation, or long-term clinical outcomes. This underscores the need for standardized vivo studies that combine Er: YAG activation with high-resolution structural and clinical endpoints.

Certain limitations of this study should be acknowledged. The experiments were conducted ex vivo, which does not fully replicate intraoral conditions such as periodontal ligament support, periapical tissues, or thermal regulation by blood flow. Specifically, ex vivo conditions do not replicate the thermal regulation provided by pulpal and periapical blood flow, nor the fluid dynamics and pressure variations present in vital teeth, which may influence both the physical effects of laser activation and the thermal response of dental tissues. Additionally, the absence of a control group limits direct comparison with alternative irrigation activation methods, though the pre-post design was appropriate for our primary objective of assessing structural safety.

Only one specific parameter combination (150 mJ, 10 Hz, 60 s) was evaluated in this study. While these parameters were selected based on demonstrated thermal safety from our previous investigation showing minimal thermal changes (< 5 °C external root surface temperature increase) under identical conditions with intracanal irrigant present, structural effects may differ with variations in power output, pulse duration, repetition rate, or tip design. Future studies should systematically evaluate multiple parameter combinations to establish a comprehensive safety window and determine optimal settings balancing antimicrobial efficacy with structural preservation.

Furthermore, this study exclusively evaluated single-rooted teeth to ensure anatomical standardization and measurement reproducibility. Multirooted teeth with complex anatomies, including isthmuses, fins, and variable canal curvatures, may respond differently to LAI, and future studies should evaluate the safety of this protocol in multirooted teeth and teeth with more complex root canal systems.

Although micro-CT provides detailed morphological analysis, scanning electron microscopy (SEM) could offer complementary insights. SEM would enable ultrastructural assessment of dentinal tubule modifications and smear layer removal beyond micro-CT’s resolution capabilities.

Future research should include in vivo studies to evaluate potential thermal effects on periapical tissues, long-term impact on dentin resistance, and comparative antimicrobial efficacy between LAI and other activation methods such as ultrasonic or sonic systems.

Within the limitations of this ex vivo study conducted on single-rooted teeth with a single set of laser parameters, Er: YAG laser activation with a side-firing spiral tip and 17% EDTA did not adversely affect dentin thickness, canal volume, or microcrack formation. These findings provide preliminary evidence supporting the structural safety of this protocol under the tested conditions and warrant further investigation through in vivo studies, comparative trials with control groups, and evaluation across a range of laser parameters and tooth types before definitive clinical recommendations can be made. Nevertheless, these results represent an important step in establishing the safety profile of this promising adjunctive irrigation activation method.

## Data Availability

No datasets were generated or analysed during the current study.
